# The efficacy of neoadjuvant immunotherapy in gastric cancer, adenocarcinoma of the esophagogastric junction, and esophageal cancer: a meta-analysis

**DOI:** 10.3389/fonc.2024.1502611

**Published:** 2024-11-22

**Authors:** Mengyi Qian, Yingying Fang, Zhiyi Xiang, Yueming Zhang, Hujie Zhan, Xiaotong Chen, Yihang Chen, Tinghui Xu

**Affiliations:** ^1^ The Second Clinical Medical College, Zhejiang Chinese Medical University, Hangzhou, China; ^2^ The First Clinical Medical College, Zhejiang Chinese Medical University, Hangzhou, China; ^3^ Intensive Care Unit, Hospital of Zhejiang People’s Armed Police, Hangzhou, China; ^4^ College of Pharmaceutical Science, Zhejiang University of Technology, Hangzhou, China; ^5^ School of Ophthalmology & Optometry, Wenzhou Medical University, Wenzhou, China; ^6^ Department of Cardiothoracic Surgery, Ningbo Yinzhou No. 2 Hospital, Ningbo, China

**Keywords:** PD-1/PD-L1 inhibitors, gastric cancer, adenocarcinoma of the esophagogastric junction, esophageal cancer, neoadjuvant immunotherapy, meta-analysis

## Abstract

**Background:**

Neoadjuvant immunotherapy holds promise in managing resectable locally advanced gastric cancer (GC), adenocarcinoma of the esophagogastric junction (AEG), and esophageal cancer (EC). However, consensus is lacking regarding the efficacy of programmed death-1 (PD-1) and programmed death ligand 1 (PD-L1) inhibitors in neoadjuvant immunochemotherapy (NICT). This study aims to assess the added benefit of PD-1/PD-L1 inhibitors in neoadjuvant chemotherapy (NCT) for these malignancies.

**Methods:**

Up to October 2024, randomized controlled trials, case-control studies, and cohort studies that evaluated the addition of PD-1/PD-L1 inhibitors to NCT were systematically retrieved from electronic databases. The primary endpoints included pathologic complete response (pCR), major pathological response (MPR), overall survival (OS), and progression-free survival (PFS).

**Results:**

Thirteen studies published between 2021 and 2024 were analyzed. Statistical analyses revealed significantly higher pCR rates (OR: 2.73, *P* < 0.001) and MPR rates (OR: 2.99, *P* < 0.001) in the NICT group compared to NCT group. The PFS was also higher in the NICT group, although the difference did not reach statistical significance (HR: 0.50, *P* = 0.072).

**Conclusion:**

This meta-analysis demonstrates that NICT enhances pathological response rates in patients with resectable locally advanced GC, AEG, and EC. However, no significant long-term prognostic benefits were associated with NICT.

**Systematic review registration:**

https://www.crd.york.ac.uk/prospero, identifier CRD42024545725.

## Introduction

Gastric cancer (GC) ranks as the fifth most prevalent malignancy worldwide and the fourth leading cause of cancer mortality (1). According to the Global Cancer Statistics Report 2022, approximately 950,000 new GC cases and nearly 700,000 deaths occur annually on a global scale (2). Similarly, esophageal cancer (EC) is the sixth leading cause of cancer death, with about 604,000 new cases and nearly 544,000 deaths reported each year (3). These cancers pose significant public health challenges, particularly in East Asian countries, where they exhibit the highest incidence rates globally.

Surgery remains the primary potentially curative treatment for resectable locally advanced GC, adenocarcinoma of the esophagogastric junction (AEG), and EC (the upper segment of EC typically managed through a combination of radiotherapy and chemotherapy). However, the five-year survival rates for GC and EC post-surgery are approximately 60% and 20%, respectively, which are unsatisfactory (4, 5). Recent evidence increasingly supports the effectiveness of neoadjuvant chemotherapy (NCT) in improving these prognoses (6, 7). Yet, the outcomes, including postoperative pathologic complete response (pCR) and long-term survival, particularly for EC, remain suboptimal, with 3-year disease-free survival (DFS) rates below 50% (8).

The role of programmed death-1 (PD-1) and programmed death ligand 1 (PD-L1) inhibitors has been established in managing unresectable or metastatic GC, AEG, and EC (9, 10). Nonetheless, their efficacy in the perioperative setting for resectable forms of these cancers remains under investigation. Several ongoing large trials, such as DANTE and NEOSUMMIT-01 for GC (11, 12), and KEEP-G03 for EC (13), aim to address these questions. Although previous meta-analyses have indicated that neoadjuvant immunochemotherapy (NICT) significantly enhances pathological outcomes, such as pCR and major pathological response (MPR), compared to conventional NCT in locally advanced EC (8, 14), the long-term prognostic impacts require further investigation due to recent updates in clinical trials. Consequently, this study aims to determine whether the addition of PD-1/PD-L1 inhibitors to NCT offers superior outcomes compared to NCT alone in patients with resectable locally advanced GC, AEG, and EC.

## Methods

### Search strategy

This meta-analysis adheres to the Preferred Reporting Items for Systematic Reviews and Meta-Analyses (PRISMA) guidelines and is registered with the international prospective register of systematic reviews (PROSPERO) under registration number CRD42024545725. We conducted comprehensive searches from the inception of the databases to October 2024 across five electronic databases: PubMed, EBSCO, Cochrane Library, Web of Science, and Embase. The searches aimed to explore the effectiveness of NICT in patients with resectable locally advanced GC, AEG, and EC. To minimize potential omissions, references from the included studies were manually searched. Search terms were developed using a combination of Medical Subject Headings (MeSH) and free-text terms as follows: (stomach neoplasms [MeSH] OR gastric cancer OR stomach cancer OR carcinoma of the stomach OR gastric carcinoma OR cancer of the stomach OR carcinoma of stomach OR cancer of the stomach OR stomach carcinoma OR esophageal neoplasms [MeSH] OR esophageal carcinoma OR esophagus cancer OR esophageal cancer OR carcinoma of esophagus OR carcinoma of the esophagus OR esophageal carcinomas OR esophagus carcinoma OR oesophageal cancer OR oesophageal carcinoma OR esophageal cancers OR gastroesophageal junction cancer OR gastroesophageal cancer OR gastro-oesophageal cancer OR gastroesophageal carcinoma OR gastro-esophageal cancer OR gastroesophageal cancers) AND (neoadjuvant therapy [MeSH] OR neoadjuvant OR neo-adjuvant therapy OR neoadjuvant chemoradiotherapy OR neoadjuvant treatment OR neoadjuvant treatments) AND (immune checkpoint inhibitors [MeSH] OR immune checkpoint blockers OR programmed death-ligand 1 inhibitors OR PD-L1 inhibitors OR nivolumab OR pembrolizumab OR dostarlimab OR durvalumab OR atezolizumab OR avelumab OR treprizumab OR sintilimab OR camrelizumab OR tremelimumab OR zimberelimab OR penpulimab OR serplulimab OR pucotenlimab OR shuglizumab OR envafolimab OR adebrelimab OR ipilimumab OR cadurizumab).

### Selection criteria

#### Inclusion criteria

Studies were included if they met all the following criteria: (1) patients diagnosed with resectable locally advanced GC, AEG, or EC; (2) the experimental group received NICT (PD-1/PD-L1 inhibitors combined with chemoradiotherapy) whereas the control group received only NCT (chemoradiotherapy); (3) reported outcomes included, but were not limited to pCR, MPR, overall survival (OS), progression-free survival (PFS), and DFS; (4) study designs were randomized controlled trials (RCTs), case-control studies, or cohort studies.

#### Exclusion criteria

Studies were excluded if they met any of the following: (1) non-English language publications; (2) lack of availability of the full text; (3) absence of accessible data; (4) studies that were superseded by more recent publications or those with larger sample sizes.

### Outcome measures

This study aims to evaluate the impact of PD-1/PD-L1 inhibitors as a form of neoadjuvant therapy on the prognosis of patients with resectable locally advanced GC, AEG, and EC. The specific outcomes measured include pCR, MPR, OS, PFS, and DFS. The pCR is defined as the absence of invasive cancer in the resected specimen, including no residual cancer in the primary tumor site or presence of only *in-situ* carcinoma. MPR is characterized by a residual tumor of 10% or less of the original tumor mass. OS measures the duration from the start of randomization to death from any cause. PFS is defined as the time from randomization to tumor progression or death from any cause, while DFS (post-surgery) measures the time from randomization to disease recurrence or death from any cause. The study explores the use of various PD-1/PD-L1 inhibitors, including pembrolizumab, socazolimab, camrelizumab, among others.

### Data extraction

Data extraction was performed independently by two researchers based on the inclusion criteria, utilizing a predefined checklist. In the event of discrepancies, the data was reviewed, and consensus was achieved through discussion. Extracted information encompassed study characteristics (authors, year of publication, sample size, country, treatment regimen, study design), patient demographics (age, gender, cancer type), and outcome metrics (pCR, MPR, OS, PFS, DFS).

### Quality assessment

Quality assessment was independently conducted by two researchers. RCTs were evaluated using the Cochrane Risk of Bias Assessment Tool, which includes criteria such as random sequence generation, allocation concealment, and blinding of participants, personnel, and outcome assessors. Cohort and case-control studies were assessed using the Newcastle-Ottawa Quality Assessment Scale, with a score of 6 or higher denoting higher quality.

### Statistical analysis

Statistical analyses were performed using STATA version 12.0 (Stata Corporation LLC, College Station, USA) and RevMan 5.3 (Cochrane Collaboration Review Manager). Dichotomous variables were assessed using odds ratios (OR) and 95% confidence intervals (95% CI). An OR greater than 1 indicated support for the experimental group, while an OR less than 1 supported the control group. Hazard ratios (HR) and 95% CI were used to evaluate long-term prognosis, with an HR less than 1 favoring the experimental group. Heterogeneity among studies was analyzed using the chi-square test and I² statistics, with I² values less than 25% indicating no heterogeneity, 25%-50% low heterogeneity, 50%-75% moderate heterogeneity, and over 75% high heterogeneity. Due to potential variability among studies, a random-effects model was applied to enhance the reliability of the findings. Subgroup analyses were conducted based on cancer type. All tests were two-sided, and a *P*-value less than 0.05 was considered statistically significant.

## Results

### Description of studies included

**Figure 1:**
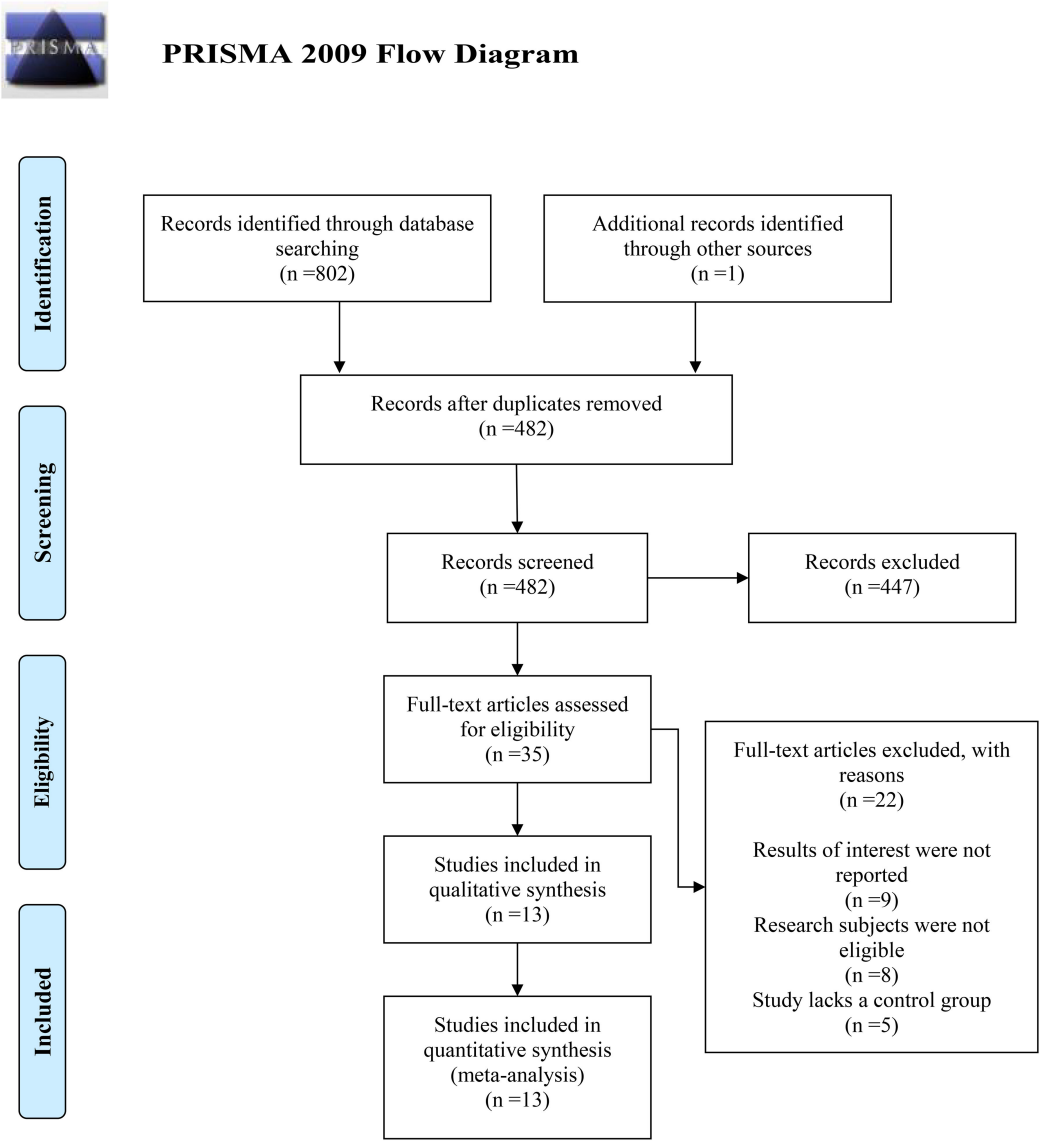
Flow diagram of selection.

A comprehensive search of five electronic databases yielded 803 search terms. After removing duplicates, 482 studies remained. Subsequent screening of titles and abstracts led to the exclusion of 447 studies due to non-compliance with the study criteria. Of the 35 studies considered further, nine were excluded for not reporting relevant outcomes, eight due to ineligible research subjects, and five due to the absence of a control group. Ultimately, 13 studies (11, 15–26) fulfilled the inclusion criteria and were analyzed (**Figure 1**).

### Characteristics of patients and trials

The 13 studies, published between 2021 and 2024, involved 2,841 participants. These studies investigated GC in four cases, GC/AEG in two, and EC in seven. The immunotherapies assessed included pembrolizumab, Atezolizumab, socazolimab, camrelizumab, among other PD-1/PD-L1 inhibitors, while the chemotherapy regimens primarily consisted of platinum and fluorouracil-based combinations. Eleven of the studies were conducted in China, one in Japan and one in German. Study designs included four RCTs, two case-control studies, and seven cohort studies. Detailed characteristics of these studies are presented in **Table 1** and **Supplementary Table S1**.

**Table 1:** Characteristics of all the studies included in the meta-analysis.

Author	Year	Number		Treatment		Types of cancer	Outcomes	Study design
		Experimental	Control	Experimental	Control			
C. Wang	2023	39	34	anti-PD1+apatinib+SOX/CAPOX	apatinib+SOX/CAPOX	GC	pCR, PFS	Cohort study
G. Xu	2024	184	184	anti-PD1/PD-L1+SOX/CAPOX/TS-1	SOX/CAPOX	GC	pCR, DFS, OS	Case-Control Study
X. Zhang	2023	34	43	anti-PD1+FLOT/SOX	FLOT/SOX	GC	pCR, MRP	Cohort study
Hui Xiong	2023	56	50	anti-PD1+apatinib+SOX/CAPOX	apatinib+SOX/CAPOX	GC	pCR, PFS, DFS, OS	Cohort study
K. Shitara	2024	502	505	pembrolizumab+cisplatin-based doublet/FLOT	placebo (saline)+cisplatin-based doublet/FLOT	gastric or gastro-oesophageal cancer	PFS, OS	RCT
Sylvie Lorenzen	2023	146	149	atezolizumab+FLOT	FLOT	gastric or gastro-oesophageal cancer	pCR	RCT
Y. Li	2023	32	32	socazolimab+nab-paclitaxel+cisplatin	placebo+nab-paclitaxel+cisplatin	EC	pCR	RCT
R.-Q. Zhou	2023	19	40	camrelizumab+docetaxel+nedaplatin	docetaxel+nedaplatin	EC	pCR	Cohort study
Y. Qiao	2022	48	206	camrelizumab+platinum-containing double-drug chemotherapy	platinum-containing double-drug chemotherapy	EC	MPR, pCR	Cohort study
B. Zhang	2003	34	97	camrelizumab+paclitaxel+platinum	paclitaxel+platinum	EC	MPR, OS	Cohort study
S. W. Jing	2022	47	47	anti-PD1+FP/TP	FP/TP	EC	pCR	Case-Control Study
B. Huang	2021	23	31	pembrolizumab+docetaxel+nidaplatin	docetaxel+nidaplatin	EC	pCR	Cohort study
Jianjun Qin	2024	130	129	camrelizumab+paclitaxel+cisplatin	paclitaxel+cisplatin	EC	pCR, MPR	RCT

GC, gastric cancer; EC, esophageal cancer; PFS, progression-free-survival; DFS, disease-free-survival; OS, overall survival; TRG, tumor regression grade; SOX, tegafur+gimeracil+oteracil+oxaliplatin; CAPOX, capecitabine+oxaliplatin; TS-1, tegafur+gimeracil+oteracil; FLOT, fluorouracil+oxaliplatin+docetaxel+calcium folinate; FP, fluorouracil+cisplatin; TP, cisplatin+paclitaxel.

### Quality assessment

Among the four included RCTs, one was classified as ‘high risk’ in other bias due to small sample size. However, all RCTs demonstrated ‘low risk’ concerning random allocation methods, allocation concealment schemes, blind methods for outcome measurers, result data integrity, and selective reporting of research results (**Supplementary Figures S1**, **S2**). Among the seven cohort studies, points were subtracted for issues such as insufficient comparability or inadequate length of follow-up, with all achieving scores of 7 or higher (**Supplementary Table S2**). Both case-control studies received a quality assessment score of 9 (**Supplementary Table S3**).

### Pathological remission rate

**Figure 2:**
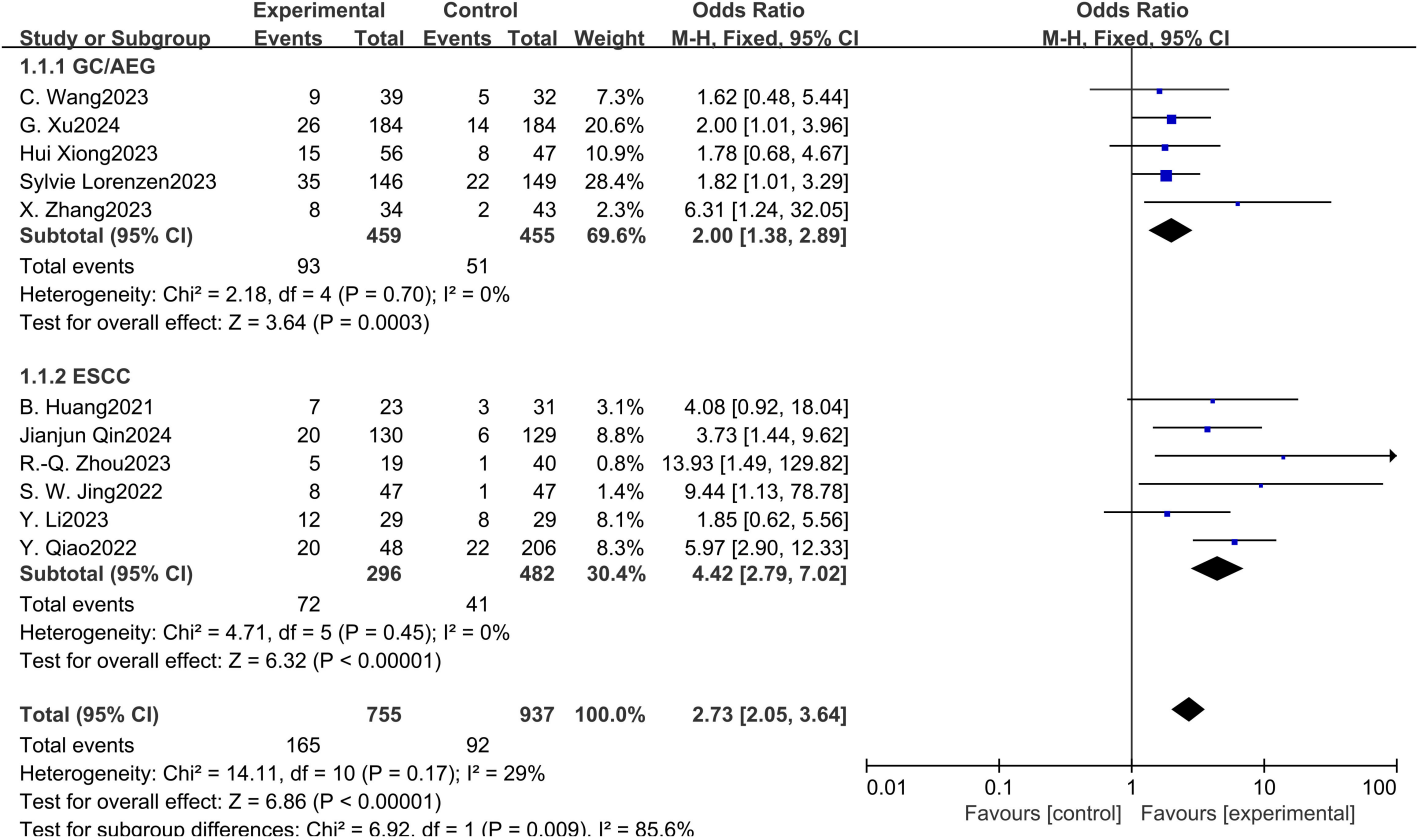
Forest plot of the pCR rate of NICT and NCT for resectable locally advanced GC, AEG, and EC.

**Figure 3:**
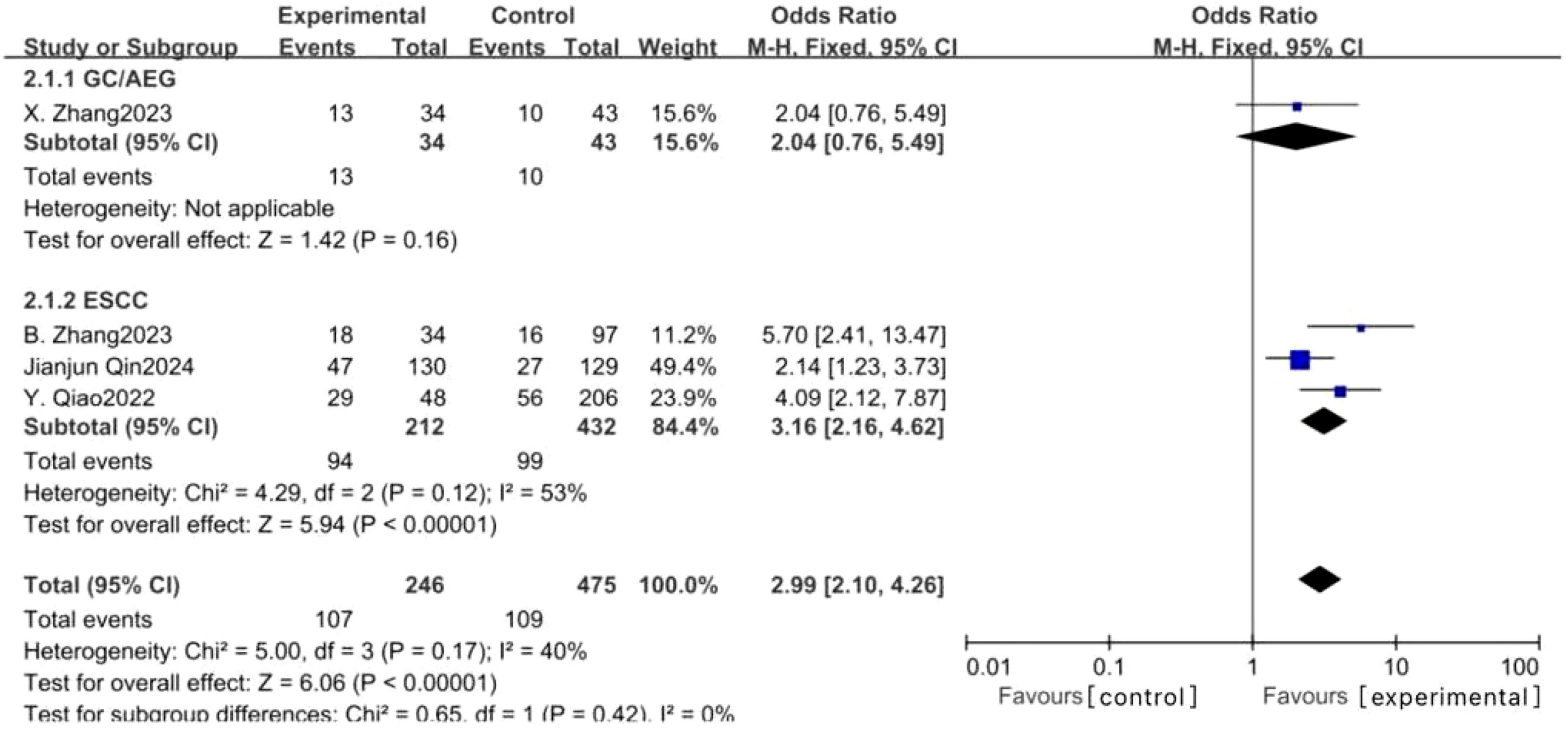
Forest plot of the MPR rate of NICT and NCT for resectable locally advanced GC, AEG, and EC.

Eleven studies examined the impact of NICT on pCR rates in patients with GC, AEG, and EC. The findings indicated a significantly higher pCR rate in the NICT group compared to the NCT group (OR: 2.73, 95% CI: 2.05-3.64, *P* < 0.001) (**Figure 2**). Analysis of five studies on GC/AEG revealed a higher pCR rate in the NICT group (OR: 2.00, 95% CI: 1.38-2.89, *P* = 0.0003). Among six studies focusing on EC, the experimental group demonstrated a greater pCR rate (OR: 4.42, 95% CI: 2.79-7.02, *P* < 0.001), suggesting a more pronounced response to neoadjuvant PD-1/PD-L1 inhibitors in EC compared to GC/AEG. Furthermore, four studies assessing MPR rates showed that NICT was more effective than NCT (OR: 2.99, 95% CI: 2.10-4.26, *P* < 0.001) (**Figure 3**). Specifically, three EC studies found a higher MPR rate in the experimental group (OR: 3.16, 95% CI: 2.16-4.62, *P* < 0.001).

### Long-term prognosis

**Figure 4:**
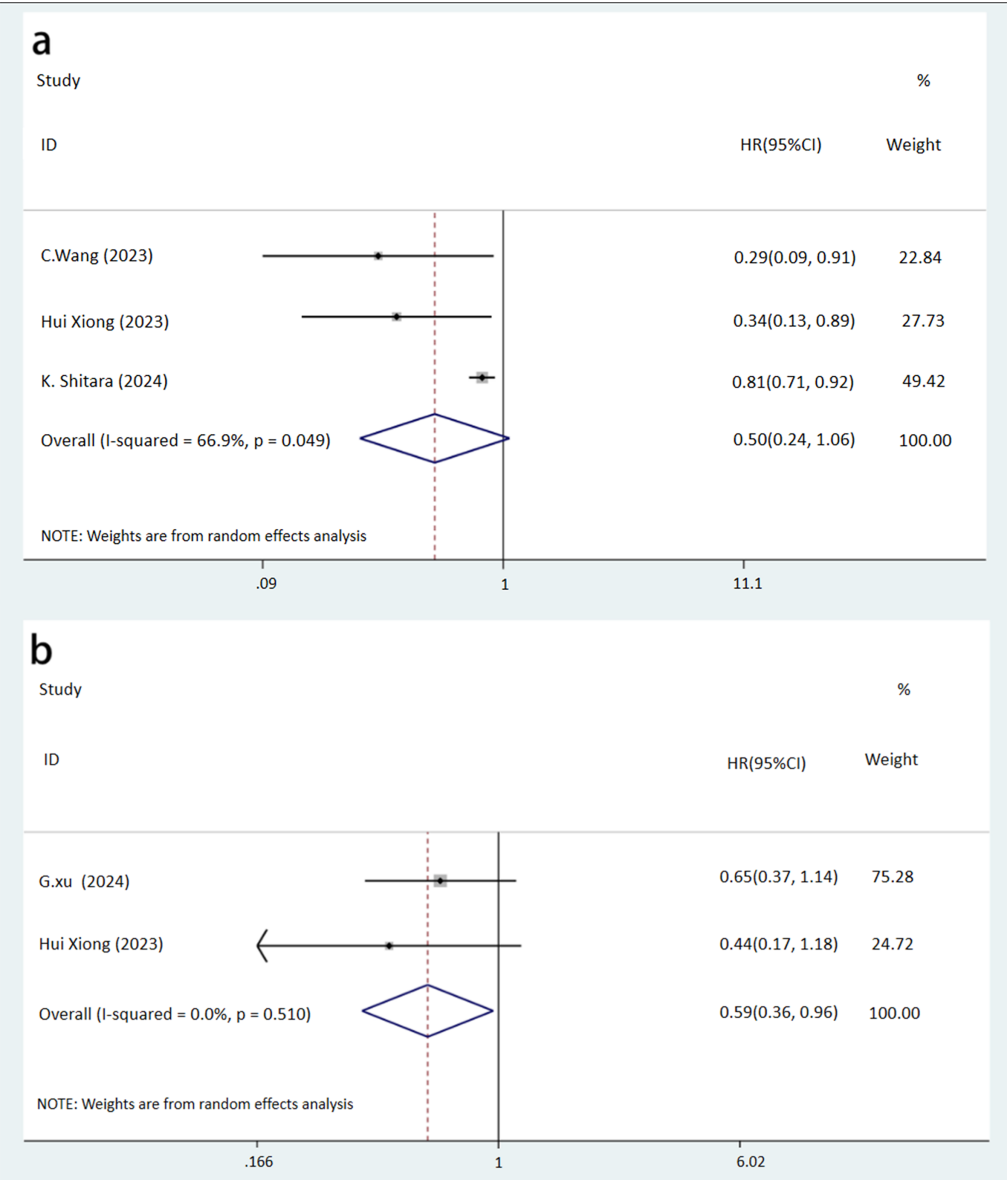
Forest plot of the prognosis of NICT and NCT for resectable locally advanced GC, AEG, and EC (**A**, PFS rate, P=0.072; **B**, DFS rate, P=0.034).

**Figure 5:**
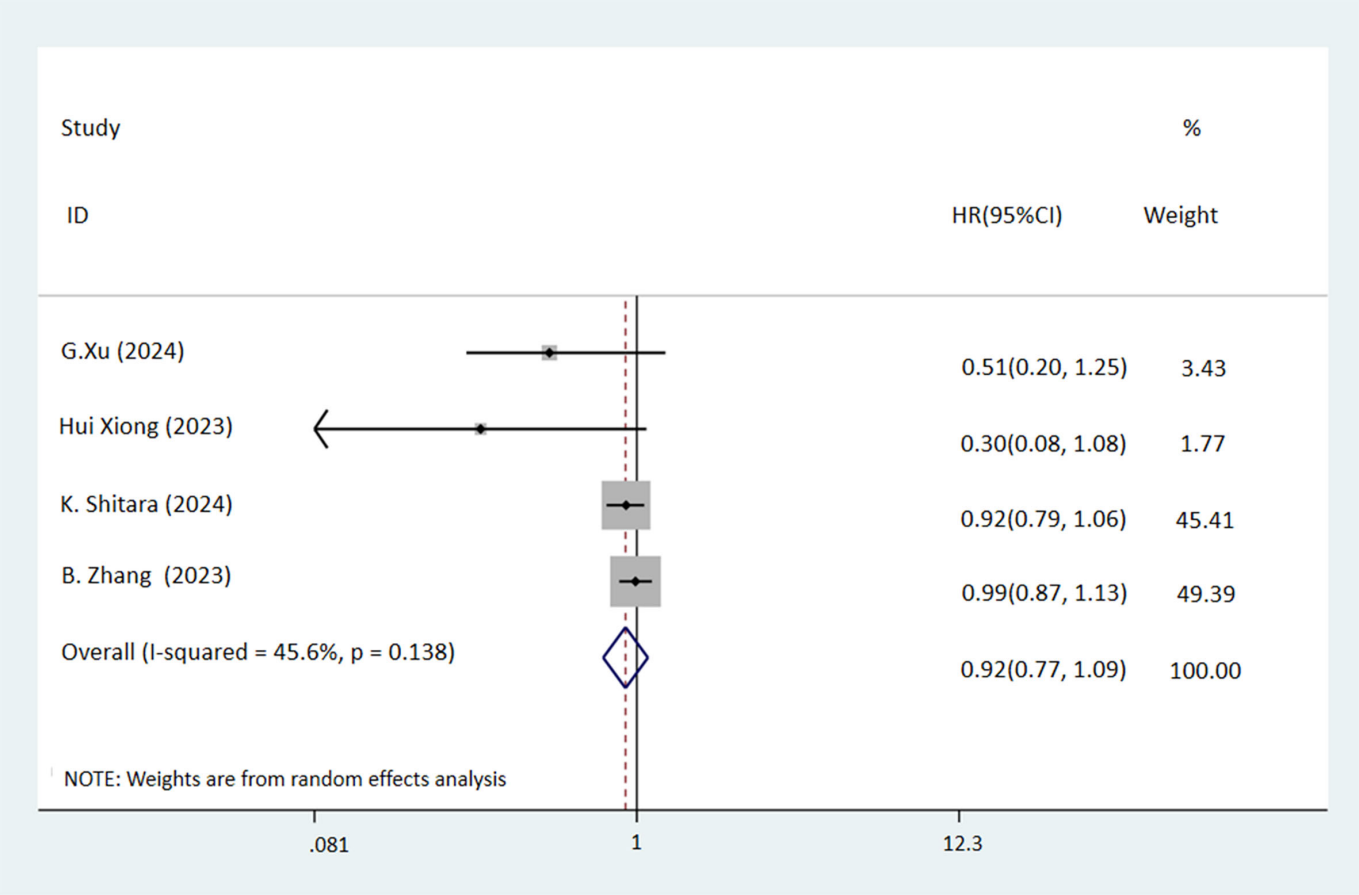
Forest plot of the OS rate of NICT and NCT for resectable locally advanced GC, AEG, and EC (P=0.324).

Analysis of PFS in three studies on GC/AEG indicated a higher rate in the NICT group, though the difference did not reach statistical significance (HR: 0.50, 95% CI: 0.24-1.06, *P* = 0.072) (**Figure 4A**). However, DFS was significantly better in the NICT group in two GC studies (HR: 0.59, 95% CI: 0.36-0.96, *P* = 0.034) (**Figure 4B**). Regarding OS in patients with GC, AEG, and EC across four studies, no significant differences were observed between the groups (HR: 0.92, 95% CI: 0.77-1.09, *P* = 0.324) (**Figure 5**).

## Discussion

Numerous studies are actively exploring the efficacy and safety of NICT in resectable locally advanced GC, AEG, and EC (17, 22). Despite this, systematic reviews that directly compare the effectiveness of NICT with conventional NCT are scarce, particularly concerning long-term prognostic outcomes. The role of PD-1/PD-L1 inhibitors in combination with NCT for these cancers remains debated. This meta-analysis aimed to assess the impact of supplementing NCT with PD-1/PD-L1 inhibitors on the long-term outcomes of patients with resectable locally advanced GC, AEG, and EC. The findings demonstrated that while NICT significantly enhanced pathological responses, such as pCR and MPR, it did not confer an advantage over NCT in terms of OS and PFS, though some improvement in DFS was noted.

The observed superior pCR and MPR rates in the NICT group compared to the NCT group can likely be attributed to the antitumor properties of PD-1/PD-L1 inhibitors. PD-1, an immune checkpoint molecule, is widely expressed across various immune cells and functions by binding to its ligand PD-L1. This interaction leads to apoptosis in cytotoxic T cells, suppression of their activation and proliferation, and prevention of autoimmune damage (27). Furthermore, PD-1 engagement recruits SHP-2, which disrupts positive signaling from the T-cell receptor and CD28, impacting downstream pathways such as PI3K-AKT and RAS-ERK (28). This modulation of T cell activity includes increasing the expression of transcription factors that counteract effector transcriptional programs and altering cellular metabolism by inhibiting glycolysis and promoting lipid catabolism and β-oxidation (29). These mechanisms collectively lead to reduced production of critical cytokines like tumor necrosis factor, interferon-gamma, and interleukin-2, facilitating immune evasion by cancer cells (30).

Additionally, PD-1 and PD-L1 expression within regulatory T cells enhances the immunosuppressive environment of the tumor microenvironment (29). PD-L1 is known to support the differentiation and functional maintenance of inducible Tregs by stabilizing Foxp3 expression and transforming naïve CD4+ T cells into Tregs through the downregulation of Akt, mTOR, and ERK2 (31, 32). Moreover, tumor-derived factors and hypoxic conditions can induce PD-L1 expression in myeloid-derived suppressor cells, further complicating the immune landscape (33).

The therapeutic application of PD-1/PD-L1 inhibitors disrupts the binding between PD-1 and PD-L1, thereby restoring T-cell functionality, which effectively targets and destroys tumor cells, enhancing pathological response. This mechanism was substantiated by the pathological outcomes observed in this study.

This study conducted subgroup analyses based on histological types of cancer, distinguishing between squamous carcinoma in EC and adenocarcinoma in GC/AEG. We found that NICT significantly improved the pCR rate across both GC/AEG and EC. These findings align with those from the meta-analysis by Deniz Can Guven et al. (34), which included seven studies on approximately 3000 patients with locally advanced non-small cell lung cancer, demonstrating a 41% reduction in disease progression or mortality and a notably higher pCR rate with NICT compared to NCT (21.8% vs. 3.8%). Similarly, Zhaoqing Tang et al. reported substantial pCR and MPR rates in locally advanced adenocarcinoma of the gastric or gastroesophageal junction, underscoring the efficacy of PD-1/PD-L1 inhibitors in these cancer types (35).

Furthermore, both adenocarcinoma and squamous carcinoma appear to benefit from PD-1/PD-L1 inhibitors (36). However, the data suggest that squamous carcinoma may derive greater benefit, which is consistent with findings by Jin Li et al. (37), who observed higher tumor mutation burdens and PD-L1 expression levels in squamous carcinoma compared to adenocarcinoma in a study involving 336 patients with cervical cancer. This was associated with increased CD4+ T-cell infiltration, highlighting a positive correlation between T-cell infiltration and immunotherapy efficacy (38). Squamous lung cancers, noted for their complex molecular features, exhibited a tumor mutational load 3.5 times greater than that of adenocarcinoma (39), suggesting a higher immunogenicity and potentially better response to immunotherapy in squamous carcinomas (40). Our study corroborates this differential response.

While numerous RCTs have shown that PD-1/PD-L1 inhibitors enhance the long-term prognosis of unresectable or metastatic GC and EC (41, 42), our findings reveal no significant differences in long-term prognostic outcomes, such as OS and PFS, for resectable locally advanced cases. This observation mirrors the results from Yoon-Koo Kang et al. (43), who found no significant enhancement in DFS with the adjunct use of PD-1/PD-L1 inhibitors alongside chemotherapy in resectable GC or AEG compared to chemotherapy alone (HR=0.90).

Consider the following factors: firstly, resectable locally advanced GC, AEG, and EC generally exhibit better prognoses than their unresectable or metastatic counterparts, particularly post-surgery. This leads to smaller survival differences between the NICT and NCT groups, making statistically significant differences less likely to emerge. For instance, the ATTRACTION-2 trial noted that the combination of chemotherapy and immunotherapy improved 2-year OS from 3.2% to 10.6% in patients with advanced GC compared to chemotherapy alone (44). However, a multicentre prospective study demonstrated that 2-year OS rates for resectable locally advanced GC were 83.0% and 90.1% for NCT and NICT, respectively (45). Our study’s findings from two GC studies suggest that NICT was more efficacious than NCT in terms of DFS, indicating that the lack of significant differences in OS and PFS may be attributed to limited sample sizes.

Secondly, Most immunotherapies aim to reactivate T-cells in the tumour, and the lymph nodes themselves are a key location for T-cells to survive and be activated. And the surgical resection of target lesions in resectable locally advanced GC, AEG, and EC, which typically includes lymph node clearance, significantly reduces lymphocyte counts. This reduction potentially undermines the efficacy of subsequent adjuvant immunotherapy (46). The findings of Matthew Spitzer et al. suggest that, based on the important role of lymphocytes in immunotherapy, consideration could be given to preserving lymph nodes for a small period of time before the end of immunotherapy (47).

Lastly, due to the large number of large-scale trials that are still open, it is not possible to access their mature data, e.g., DANTE (11), MATTERHORN (48), Dragon-IV (49). These studies have large sample sizes and high confidence in their data. Their results may point to a survival benefit from NICT.

The principal strength of this study lies in its focus on the long-term prognostic effects of NICT on resectable locally advanced GC, AEG, and EC, providing valuable insights into the potential benefits of adding PD-1/PD-L1 inhibitors to NCT. The inclusion of updated RCTs conducted between 2021 and 2024 ensures that the results are relevant to current therapeutic practices.

However, this study also has limitations. The inclusion of observational studies potentially reduces the overall level of evidence. The presence of heterogeneous tumor types and study designs necessitated the application of a random effects model to manage variability. Furthermore, the limited number of studies included restricted the ability to perform extensive subgroup analyses. Finally, since the study population was exclusively Asian, the findings may not be directly generalizable to other demographic groups.

## Conclusion

This meta-analysis substantiates the efficacy of NICT in enhancing pathological responses, specifically pCR and MPR, in patients with resectable locally advanced GC, AEG, and EC. However, it did not demonstrate a benefit in long-term prognostic outcomes such as OS and PFS. These results suggest avenues for future research, emphasizing the need for larger multicentre RCTs to corroborate and refine these findings.

## Data Availability

The original contributions presented in the study are included in the article/**Supplementary Material**. Further inquiries can be directed to the corresponding author.

## Supplementary material

Supplementary Figure 1 Proportion of risk of bias based on the evaluation domains listed in the Cochrane Collaboration Risk of Bias Tool.

Supplementary Figure 2 Summary of the risk of bias based on the evaluation domains listed in the Cochrane Collaboration Risk of Bias Tool.
