# Supplementary figures and images for: The efficacy of neoadjuvant immunotherapy in gastric cancer, adenocarcinoma of the esophagogastric junction, and esophageal cancer: a meta-analysis
Source: Front Oncol. 2024 Nov 22;14:1502611. doi: 10.3389/fonc.2024.1502611 (PMC11621004; doi:10.3389/fonc.2024.1502611)

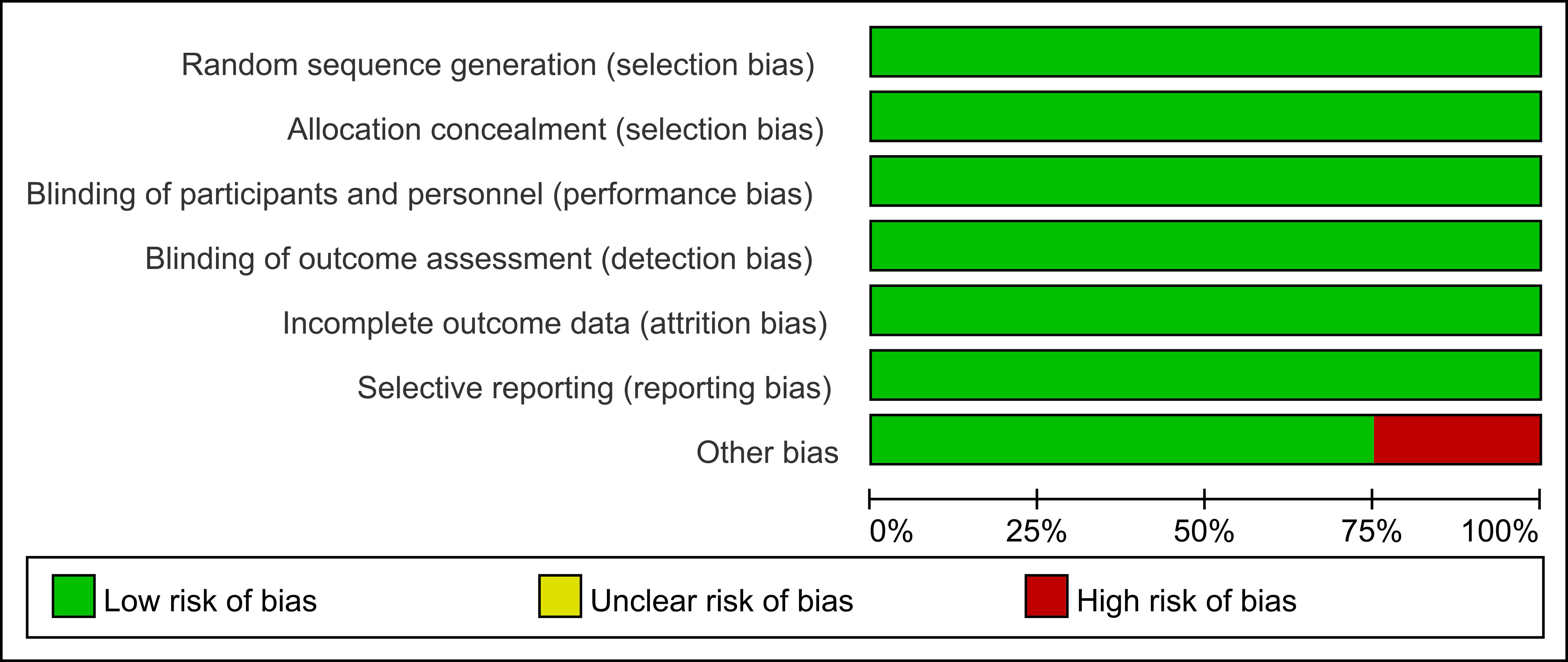

Supplement: Supplementary Figure 1 — Proportion of risk of bias based on the evaluation domains listed in the Cochrane Collaboration Risk of Bias Tool. [file Image1.tif]

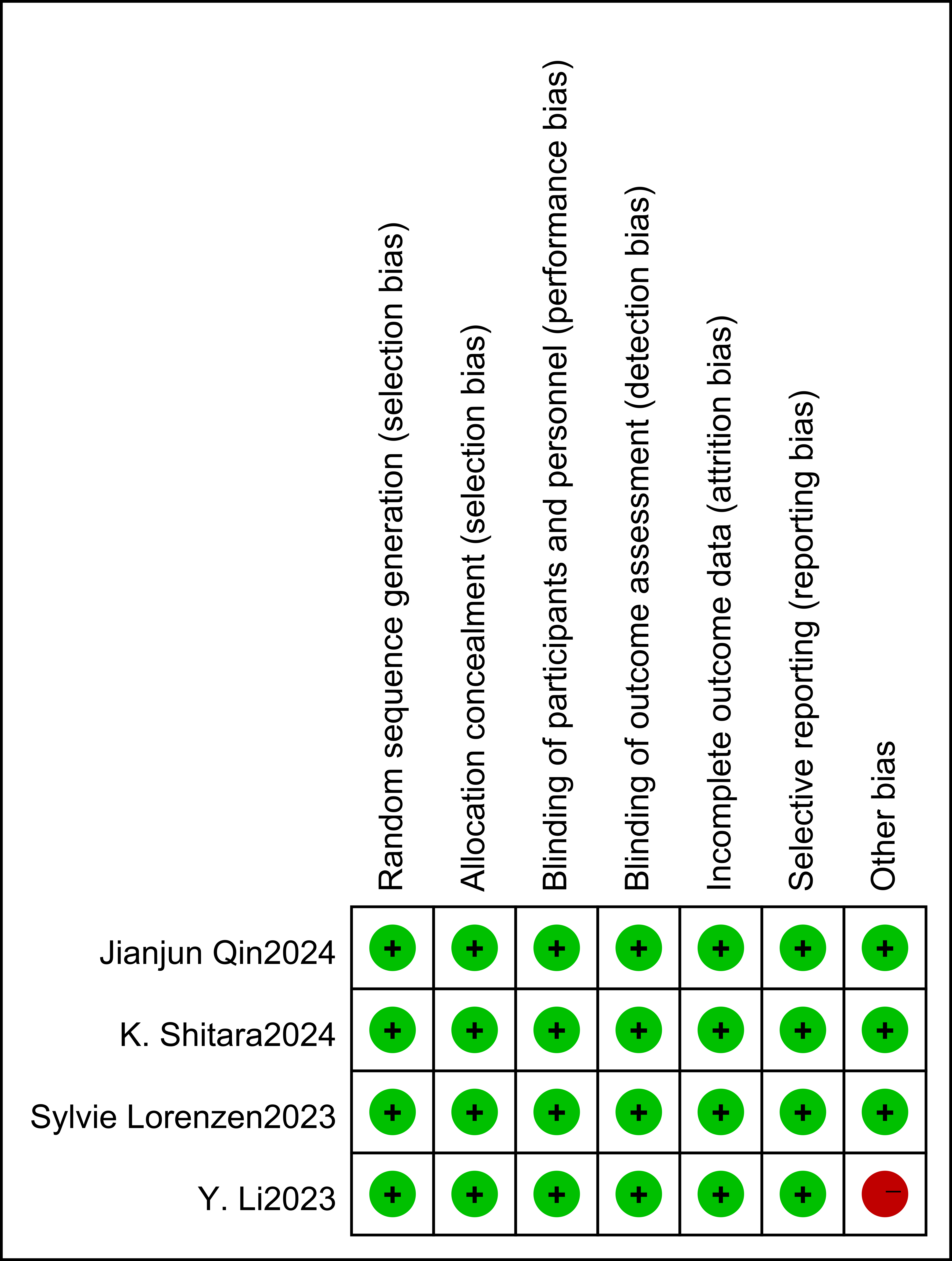

Supplement: Supplementary Figure 2 — Summary of the risk of bias based on the evaluation domains listed in the Cochrane Collaboration Risk of Bias Tool. [file Image2.tif]
