# Supplementary material for: The efficacy of neoadjuvant immunotherapy in gastric cancer, adenocarcinoma of the esophagogastric junction, and esophageal cancer: a meta-analysis
Source: Front Oncol. 2024 Nov 22;14:1502611. doi: 10.3389/fonc.2024.1502611 (PMC11621004; doi:10.3389/fonc.2024.1502611)
Supplement: Supplementary file 3 [file Table1.docx]

**Supplementary Table 1. Characteristics of all the studies included in the meta-analysis.**

| Author | Year | Country | Stage | Age (year) | | Proportion of males(%) | | Median follow-up (month) | Surgery | |
| --- | --- | --- | --- | --- | --- | --- | --- | --- | --- | --- |
|  |  |  |  | Experimental | Control | Experimental | Control |  | Experimental | Control |
| C. Wang | 2023 | China | IIb–IIIc | 57.9±9.1 | 58.9±10.2 | 25 (64.1%) | 25 (73.5%) | 23 | 39 (100%) | 32 (94.1%) |
| G. Xu | 2024 | China | IIa-IVa | 62.50 (54.00, 68.00) | 62.00 (54.00, 67.00) | 145 (78.80%) | 148 (80.43%) | 9 | 184 (100%) | 184 (100%) |
| X. Zhang | 2023 | China | II-III | 60.1±11.4 | 59.1±9.1 | 26 (76.5%) | 36 (83.7%) | 22 | 34 (100%) | 43 (100%) |
| Hui Xiong | 2023 | China | IIb–IIIc | 58.2±8.7 | 59.1±9.9 | 37 (66.1%) | 37 (74%) | / | 56 (100%) | 47 (94.0%) |
| K. Shitara | 2024 | Japan | II–IVa | 64 (56–70) | 63 (55–69) | 288 (72%) | 287 (71%) | 48 | 428 (85.3%) | 421 (83.4%) |
| Sylvie Lorenzen | 2023 | German | IIa–IVa | 61 (29-79) | 62 (23-80) | 116 (80%) | 101 (68%) | / | 141 （ 97%） | 143 （ 96%） |
| Y. Li | 2023 | China | IIa-IIIc | 61.0 (53–72) | 63.0 (47–74) | 23 (71.9%) | 28 (87.5%) | / | 29 (90.6%) | 29 (90.6%) |
| R.-Q. Zhou | 2023 | China | I–IVa | 65.89±6.06 | 64.50±4.54 | 17 (89.5%) | 31 (77.5%) | / | 19 (100%) | 40 (100%) |
| Y. Qiao | 2022 | China | Ia-IIIc | 64.15 ± 7.293 | 62.22 ± 7.136 | 38 (79.2%) | 147 (71.4%) | / | 48 (100%) | 206 (100%) |
| B. Zhang | 2003 | China | Ib–IIIc | 60.68 ± 7.44 | 60.08 ± 7.78 | 31 (91.2%) | 94 (96.9%) | 20 | 34 (100%) | 97 (100%) |
| S. W. Jing | 2022 | China | II–IVa | / | / | 30 (63.8%) | 33 (70.2%) | / | 47 (100%) | 47 (100%) |
| B. Huang | 2021 | China | II–IVa | 59.2±7.3 | 58.9±6.4 | 21 (91.3%) | 30 (96.7%) | / | 21 (91.3%) | 27 (87.1%) |
| Jianjun Qin | 2024 | China | Ib–IVa | 63（ 44 - 75） | 65（ 44 - 75） | 112 (86.2%) | 104 (80.6%) | 8.2 | 116(89.2%) | 103(79.8%) |
