# Supplementary material for: The efficacy of neoadjuvant immunotherapy in gastric cancer, adenocarcinoma of the esophagogastric junction, and esophageal cancer: a meta-analysis
Source: Front Oncol. 2024 Nov 22;14:1502611. doi: 10.3389/fonc.2024.1502611 (PMC11621004; doi:10.3389/fonc.2024.1502611)
Supplement: Supplementary file 4 [file Table2.docx]

**Supplementary Table 2.** Quality assessment of cohort studies included.

| Author, year | **Selection (Out of 4)** | | | | **Comparability**  **(Out of 2)** | **Outcomes (Out of 3)** | | | **Total**  **(Out of 9)** |
| --- | --- | --- | --- | --- | --- | --- | --- | --- | --- |
|  | Representativeness of exposed cohort | Selection of non exposed cohort | Ascertainment  of exposure | Outcome not present at the start of the study |  | Assessment of outcomes | Length of follow-up | Adequacy of follow up of cohorts |  |
| C. Wang. 2023 | 1 | 1 | 1 | 1 | 2 | 1 | 1 | 1 | 9 |
| X. Zhang. 2023 | 1 | 1 | 1 | 1 | 1 | 1 | 1 | 1 | 8 |
| Hui Xiong. 2023 | 1 | 1 | 1 | 1 | 1 | 1 | 0 | 1 | 7 |
| R.-Q. Zhou. 2023 | 1 | 1 | 1 | 1 | 1 | 1 | 0 | 1 | 7 |
| Y. Qiao. 2022 | 1 | 1 | 1 | 1 | 2 | 1 | 0 | 1 | 8 |
| B. Zhang. 2023 | 1 | 1 | 1 | 1 | 2 | 1 | 1 | 1 | 9 |
| B. Huang. 2021 | 1 | 1 | 1 | 1 | 1 | 1 | 0 | 1 | 7 |

The cohort studies were assessed by the Newcastle-Ottawa Quality Assessment Scale (NOS) checklist.
