# Supplementary material for: The efficacy of neoadjuvant immunotherapy in gastric cancer, adenocarcinoma of the esophagogastric junction, and esophageal cancer: a meta-analysis
Source: Front Oncol. 2024 Nov 22;14:1502611. doi: 10.3389/fonc.2024.1502611 (PMC11621004; doi:10.3389/fonc.2024.1502611)
Supplement: Supplementary file 5 [file Table3.docx]

**Supplementary Table 3.** Quality assessment of case-control studies included.

| Author, year | **Selection (Out of 4)** | | | | **Comparability**  **(Out of 2)** | **Outcomes (Out of 3)** | | | **Total**  **(Out of 9)** |
| --- | --- | --- | --- | --- | --- | --- | --- | --- | --- |
|  | Adequate case definition | Representativeness of the cases | Selection of controls | Definition of controls |  | Ascertainment of exposure | Same method of ascertainment for cases and controls | Non-response rate |  |
| G. Xu. 2024 | 1 | 1 | 1 | 1 | 2 | 1 | 1 | 1 | 9 |
| S. W. Jing. 2022 | 1 | 1 | 1 | 1 | 2 | 1 | 1 | 1 | 9 |

The case-control studies were assessed by the Newcastle-Ottawa Quality Assessment Scale (NOS) checklist.
